# The Development and Validation of a Rapid Assessment Tool of Primary Care in China

**DOI:** 10.1155/2016/6019603

**Published:** 2016-01-17

**Authors:** Jie Mei, Yuan Liang, LeiYu Shi, JingGe Zhao, YuTan Wang, Li Kuang

**Affiliations:** ^1^Institute of Social Medicine and Health Management, School of Public Health, Sun Yat-sen University, 74 Zhongshan 2nd Road, Guangzhou, Guangdong 510080, China; ^2^Johns Hopkins Bloomberg School of Public Health, Johns Hopkins Primary Care Policy Center, 624 North Broadway, Baltimore, MD 21205, USA

## Abstract

*Introduction*. With Chinese health care reform increasingly emphasizing the importance of primary care, the need for a tool to evaluate primary care performance and service delivery is clear. This study presents a methodology for a rapid assessment of primary care organizations and service delivery in China.* Methods*. The study translated and adapted the Primary Care Assessment Tool-Adult Edition (PCAT-AE) into a Chinese version to measure core dimensions of primary care, namely, first contact, continuity, comprehensiveness, and coordination. A cross-sectional survey was conducted to assess the validity and reliability of the Chinese Rapid Primary Care Assessment Tool (CR-PCAT). Eight community health centers in Guangdong province have been selected to participate in the survey.* Results*. A total of 1465 effective samples were included for data analysis. Eight items were eliminated following principal component analysis and reliability testing. The principal component analysis extracted five multiple-item scales (first contact utilization, first contact accessibility, ongoing care, comprehensiveness, and coordination). The tests of scaling assumptions were basically met.* Conclusion*. The standard psychometric evaluation indicates that the scales have achieved relatively good reliability and validity. The CR-PCAT provides a rapid and reliable measure of four core dimensions of primary care, which could be applied in various scenarios.

## 1. Introduction

Primary care is a fundamental part in the health care systems of both high and low income countries and there is ample evidence that primary care is closely related to the improvement of health outcomes [1, 2]. In 1994, the Institute of Medicine (IOM) proposed a function-orientated definition of primary care, which is consistent with the widely acknowledged multidimensional concept of primary care with its emphasis on the four core dimensions of primary care (i.e., first contact, continuity, comprehensiveness, and coordination) [3–5].

In urban China, primary care is defined as “the delivery of comprehensive, continuous, and convenient episodic and preventive health care services to families in the community” [6], which are mostly provided by general practitioners (GPs) in community health centers (CHCs) or other health stations or clinics [7]. Since 2009, the Chinese central government has launched a new health care reform, an essential part of which is to strengthen the primary care system [8–10]. Significant government investments have been made on developing GP workforce, constructing CHCs infrastructure, improving CHCs distribution network in order to improve the geographic accessibility, providing 12 basic public health services in CHCs, and so on [9, 11]. Meanwhile, the social health insurance have covered approximately 95% of the population and its reimbursement strategy has strengthened the use of primary care and the promotion of CHCs as a potential gatekeeper [12–14].

As an economically developed district in southern China, Guangdong province has long recognized the importance of primary care in promoting health for the general public and has launched pilot programs for regional primary care reform since 1996 [15]. There are some favorable changes; for example, general practitioners (GPs) who are at the frontier in providing primary care have gradually acquired a good local reputation and attracted large numbers of patients. In the meantime, a notable characteristic of the province is that various management patterns of CHCs emerged after years of reform and the performance of CHCs varied under different management patterns (discussed later).

Whilst there has been considerable government investment and policy attention on primary care, evidence of the quality of primary care services is urgently needed [10, 16]. Previous researches in China have been carried out to assess the quality of primary care in disease-focused or task-orientated manners [17–20]. Although these studies have been essential, they largely fell short on evidence of the core dimensions of primary care. A previous systematic review has demonstrated that the four core dimensions determine the primary care process [21]. Primary care is deemed stronger with better fulfillment of these dimensions [22]. Therefore, it is reasonable and necessary to develop a valid tool to measure the quality of primary care in a multidimensional manner and include its four core dimensions [23]. Several studies in developed countries have successfully developed valid tools in this way [24–27] and such evaluation tools have been used in China before [7, 28]. Our preliminary research tried out such tools in practice. However, the results indicated that they were time-consuming and generated a significant amount of “not sure/don't remember” responses. Therefore, constructing a rapid assessment tool seems imperative so that the Chinese primary care evaluation tool becomes concise and user-friendly in order to obtain key information with minimum time cost as well as to ensure patient compliance and quality of data.

This study was undertaken in close collaboration with health authorities of Guangdong province in response to the need for a rapid assessment tool to measure the quality of primary care service delivery. The objective of this paper was first to construct a rapid assessment tool that was based on the core dimensions of primary care and, secondly, to apply analytic methods to assess the validity and reliability of this tool. In addition, analysis was undertaken to compare primary care achievements across different health care settings.

## 2. Methods

### 2.1. Instrument

Primary Care Assessment Tool (PCAT) has been developed based on the theoretical model of primary care attributes established by John Hopkins [29] and Donabedian's quality framework [30]. PCAT has been highly recommended over other measurement tools when assessing the process of primary care and is available in various formats [26, 30–32]. Primary Care Assessment Tool-Adult Edition (PCAT-AE) is designed to measure adult patients' experience of primary care. It has originally been used in the US [26] and has been adopted in several other countries with different health systems, including Brazil [33], Korea [34], Spain [35], and China [7, 28]. After certain adaptions, all these tools had been validated in their corresponding countries, and some of them have been used to measure quality of primary care [33, 36, 37].

In our study, the translation and adaption of PCAT-AE involved several steps. First, the questionnaire was translated into Chinese. Second, a group of four general practitioners and two experts of primary care policy reviewed the translated PCAT and some of the items that did not fit in the Chinese context were rephrased. During this stage, any item that did not adapt to the Chinese context, shared similar conceptual basis, or lacked conciseness was rewritten, combined, or deleted. Modifications were only made on condition that a consensus was reached; for example, “home safety, such as getting and checking smoke detectors and storing medicines safely” was eliminated, because it was considered to be inappropriate or unpractical in the current Chinese context; “When your PCP is closed and you get sick during the night, would someone from there see you that night” was deleted since most of the CHCs in China do not offer medical services at night. Then pilot tests, which focused on item wording, were conducted in three facilities. Twenty patients were randomly selected to complete the questionnaire by themselves in each of the pilot test and items were further revised into more understandable words to ensure that elderly or subjects of lower socioeconomic status can fully comprehend (specific item changes can be found in Table 9).

In this stage, the Chinese Rapid Primary Care Assessment Tool (CR-PCAT) consisted of 42 items. 23 items assessed the four core dimensions of primary care—first contact, continuity, comprehensiveness, and coordination—and three derivative dimensions—family centeredness, community orientation, and cultural competence. Two items were developed to identify individuals' usual source of care and another 2 measured the frequency of visits to the general practitioners while the rest of the items mainly reflected patients' social demographic information. The services received by patients were represented by a 4-point Likert scale (1 = never; 2 = sometimes; 3 = often; 4 = always). An additional option of Don't know/Not sure was added in case of lack of knowledge of a certain item. The Don't know/Not sure response and missing data were assigned a neutral value of 2.5 when conducting the analyses to be consistent with the methods used in other countries [34, 35].

### 2.2. Data Collection

A stratified, three-stage sampling approach was used to decide study sample. Three cities, namely, Guangzhou, Shenzhen, and Dongguan, were chosen, since they displayed significant socioeconomic variations and also CHCs were under different management pattern in these cities. In Guangzhou, 90% of the CHCs were owned and operated by government, and the remaining 10% were private-owned. The study chose one government-owned CHC and one private-owned CHC that were both granted as a state-level demonstration site, and another ordinary government-owned CHC. In Dongguan, all CHCs were government-owned and government-run; one state-level demonstration site along with one ordinary CHC was selected. In Shenzhen, all CHCs were affiliated with local hospitals. We selected two state-level demonstration sites and one ordinary CHC. Convenience sampling was then used to select patients in each of the CHC to participate in the survey.

The standard sample size formula for cross-sectional survey was used to calculate the sample size, where *Z*_1−*α*/2_ = standard normal variant corresponding to 5% type I error of 1.96; *p* = expected proportion in population based on previous study; *d* = absolute error or precision [38]. An estimated of the proportion was drawn from previously published research [7]. Typically, a conservative estimate of the proportion (i.e. 50/50) was made, if data was not sufficient. In addition, the sample size was adjusted for refusal rate of approximately 10%. Finally, a target sample size of 400 was set within each area. The CHCs in Guangzhou were oversampled because of additional planned analysis (not the focus of this paper). Furthermore, expert consultation was also used to ensure the precision and feasibility of the sample size, which confirmed the calculation above.

Data collection began in June 2014 and lasted for three months. Interviewers who were postgraduate students from Sun Yat-sen University were trained by two researchers in advance in order to assist the patients to complete the questionnaires. One-to-one interviews were conducted to guarantee the quality of survey data. Adult patients who were 18 years or older and could speak Mandarin or Cantonese were selected at the waiting area of each site. Subjects selected were exclusively to patients who were visiting general practitioners. Those who had visited the same general practitioners at least three times were eligible since they were considered to have a better understanding of primary care services provided by GPs. These patients were asked for permission to participate in the interview with a full explanation of the research purpose and were told that the survey would not influence their GP visits. Patients who took part in the survey were given a small gift as a token of appreciation upon completion of the questionnaire.

### 2.3. Data Analysis

Data analysis consisted of description of the sample population, validation of the CR-PCAT, and descriptive summary of the primary care achievement of each city. First, socio-demographic characteristics and health data of the sample population were summarized to demonstrate a diverse sample of populations and their utilization of primary care, meanwhile, the characteristics of each city and CHC were also presented.

Second, validation of the CR-PCAT was conducted, which was applied exclusively to the 23 items evaluating the seven dimensions of primary care [39]. For construct validity, factor analysis (principal component analysis and varimax rotation) was undertaken to test the dimensionality of the hypothesized scales. The item selection criteria were: eigenvalues >1.0, factor loading >0.35, and all retained items should not have a secondary loading over 0.35. Then internal consistency reliability of each scale was assessed by Cronbach's coefficient alpha (*α*) and item-total correlation. Cronbach's coefficient alpha over 0.50 was recommended as the benchmarks of adequacy of reliability coefficients [40–42], while the minimum acceptable item-total correlation was 0.30 [41].

Next, five Likert scaling assumptions were tested, which included (1) item-convergent validity (a test by item-scale correlations); (2) item-discriminant validity (a test by the scaling success rate, i.e. items within a scale correlate more substantially with their hypothesized scale than with any other scale); (3) equal item variance (a test by examining item means, standard deviations, the equivalence of the intra-class correlation and Scotts homogeneity ratio for each scale); (4) equal item-scale correlation (a test by examining the range of item-scale correlations); and (5) score reliability (a test by Cronbach's coefficient alpha) [43, 44]. The analysis was only applied to the retained items.

Then, we examined the following score distribution characteristics of the revised scales: mean, standard deviation, quartiles, skewness, range, and Kurtosis. Each of the five scales was also assessed for inter-factor correlations by computing a matrix of inter-factor correlations [45]. Finally, primary care achievement of different cities was compared using analysis of variance.

All analyses were performed using the statistical package STATA 13.

## 3. Results

### 3.1. Subjects

A total of 1831 eligible individuals were asked to participate in the survey, and 81.9% (*n* = 1499) of them completed the questionnaire. After eliminating samples with more than 5 missing responses (*n* = 35), 1465 effective samples were included for analysis.

Table 1 shows the socio-demographic features and health care utilization of the survey participants. The analytic sample included adults aged 18 to 89 years (mean = 46.1 years). They were predominantly female (60.7%), and were resident or temporary resident population (98.6%) with generally lower level of household income (only 10.9% over 10000 yuan/month). Over half of respondents were employed (54.6%) with relatively lower education (69.1% did not finish high school). Approximately one third (41.8%) indicated having chronic disease and 23.3% rated their health as fair or bad. Most participants (78.7%) were covered by health insurance and had been visiting their PCP for more than 1 year (82.3%). 73.3% reported over 3 times of visits to their PCP and over half of the respondents had visited a specialist in the past year (53.4%). Less than one tenth of all users had signed contracts with their PCP in spite of government's vigorous promotion (8.9%) [46].

Table 2 displays the characteristics of the included cities and CHCs. Of the eight CHCs being studied, one CHC from Guangzhou was private-owned with the other 7 owned by the local government. Gatekeeping system was not implemented in Guangzhou, while in Dongguan and Shenzhen it has been implemented in practice. It is also worth noting that only the patients who were enrolled in a specific insurance plan were under the impact of the gatekeeping system (not the focus of this paper). Of the three CHCs in Shenzhen, they were all affiliated with the local public hospitals while CHCs in the other two cities were operated independently.

### 3.2. Construct Validity and Reliability

Twenty-three items were included in the initial principal component analysis. Based on the criteria that eigenvalue was larger than 1.0, five components that corresponded to the four core dimensions of primary care remained: first contact utilization, first contact accessibility, ongoing care, comprehensiveness, and coordination (Table 3). These extracted factors explained 58.91% of the total variance, with eigenvalues ranging from 2.08 to 1.39.

Eight of the 23 items were eliminated based on the criteria imposed for factor analyses (Table 4). No items were deleted for first contact utilization, ongoing care, and comprehensiveness. One item was eliminated for first contact accessibility and coordination dimension, respectively. Although first contact accessibility dimension contained only two items after elimination, it was retained since it represented unique conceptual meaning of primary care. All items were deleted for family centeredness, community orientation, and cultural competence.

Table 4 presents the item descriptive results, as well as results of reliability tests for both the original items and the final items. The distribution of the items varied significantly from a mean of 1.09 (When your PCP is closed, can you get advice quickly over the phone if you need it?) to 3.63 (When you have a new health problem, do you go to your PCP before going somewhere else?) on the 4-point Likert scale. The distribution tends to skew toward less favorable answers, with only five items falling above 2.5. The first contact utilization and ongoing care scales achieved the highest mean scores, whereas scales with lower means were first contact accessibility, comprehensiveness, and coordination.

Reliability tests include item-total correlation and Cronbach's alpha coefficient reliability. Item-total correlations show that each item was strongly associated with its corresponding scale, with value ranging from 0.56 (When you have a new health problem, do you go to your PCP before going somewhere else? and When your PCP is closed, can you get advice quickly over the phone if you need it?) to 0.83 (When you go to your PCP's, are you taken care of by the same doctor or nurse each time?). Cronbach's alpha demonstrates internal consistency reliability that was higher than or equal to the original scales, despite dropping items from original scales.

### 3.3. Testing the Likert Scaling Assumptions

Table 5 demonstrates a summary of the results of the tests of Likert scaling assumptions using the five multi-item revised scales. All item-scale correlations exceeded 0.5 with the majority greater than 0.6 and all scales demonstrated a relatively narrow range of item-scale correlations (from 0.04 for first contact accessibility to 0.19 for first contact utilization). All 5 multi-item scales achieved 100% scaling success, indicating that all items in these scales got a greater correlation with items in their hypothesized scale than with items in other scales. Formal evidence of equal item variance was supported by the proximity of the intraclass correlation and Scotts homogeneity ratio for each scale. Two multi-item scales achieved Cronbach's alpha level over 0.70, and two scales were below the threshold, but exceeded 0.50. One scale, first contact utilization, was far below the standard, verifying the low homogeneity of variances among items within the scale (0.2). However, the scale was retained because of their conceptual significance.

### 3.4. Descriptive Feature of CR-PCAT

Table 6 presents estimates of central tendency, dispersion, and other features of scale score distributions for the five primary care scales. The full range of possible scores was observed for all scales. Except for first contact utilization and ongoing care, the other three scales were positively skewed, indicating distributions with more negative ratings of primary care. The percentage of respondents scoring at the floor (the lowest score) or ceiling (the highest score) was acceptably low for all scales. The most significant floor effects existed in the first contact accessibility scale where over 80% of the participants reported minimum score.

Table 7 compares the internal reliability coefficient and interfactor correlation for each primary care scale. Cronbach's alpha of each scale was substantially greater than its correlation with all other primary care scales. None of the interfactor correlations were excessively high, indicating the uniqueness of each primary care scale. All significant correlations were positive, implying that each scale was complementary to some extent. Relatively high and positive interfactor correlations were observed between first contact utilization and coordination (0.32), with the latter and comprehensiveness (0.27), and with comprehensiveness and ongoing care (0.29).

### 3.5. Primary Care Achievements in Three Cities

Table 8 illustrates that the overall scores of primary care achievements were relatively low in general, with Shenzhen achieving the highest total score (2.19), followed by Dongguan (2.10), and Guangzhou achieving the lowest score (2.09). For each scale, Guangzhou got the highest scores on two scales (ongoing care and comprehensiveness) and Shenzhen on first contact accessibility and coordination while Dongguan achieved the highest score on one scale (first contact utilization). Besides, most of the scores of each scale differed significantly among the three cities.

## 4. Discussion

The CR-PCAT introduced in this paper is an explicit and brief adaptation of the original PCAT-AE to the Chinese context. The final version of CR-PCAT comprises 15 items with five scales measuring four core dimensions of primary care. The core dimensions and items in corresponding dimensions display high consistency with the original PCAT-AE.

A standard psychometric evaluation method was used to validate the CR-PCAT. The analytic results indicate that the hypothesized scales have achieved relatively good reliability and validity. Principal component factor analysis supports the scale's construct validity and the extracted five factors explained 58.91% of the total variance in the item scores. The panel of experts in primary care helped facilitate the best possible content validity [39]. Reliability tests indicated adequate item-total correlation and internal consistency of the tool. All of the five Likert scaling assumptions, including item-convergent validity, item-discriminant validity, equal item variance, equal item-scale correlation, and score reliability, were basically met, which suggests the appropriateness of the usage of Linker's method.

The three derivative dimensions—family centeredness, community orientation, and cultural competence—were eliminated, because retaining all these three derivative dimensions did not increase the explained variations. Besides, the items within the three dimensions did not converge together as the other studies had displayed [26]. To balance between the amounts of information captured and time costs, only the four core dimensions with five scales were retained, which also met our goal of developing a compact measure tool. In addition, we also ran factor analysis using data from each of the three cities separately. The results confirmed the validity of the factor analysis done with combined dataset (results are not shown due to large amount of information but are available upon request).

Item descriptive results indicated that most of the items scores skew toward less favorable answers, which contrasted with the results of other studies [7, 34]. High rate of favorable response and “Don't know/Not sure” answers was common among Asian populations, since they were not used to commenting or critiquing. However, the study adopted one-to-one interview to ensure that all participants fully understood each item and reported their actual perception of primary care.

### 4.1. Application of the Tool

CR-PCAT has multiple uses given its small number of items. Compared to the PCAT-AE and the other version of PCAT, which needs approximately 20–40 minutes to complete [26], the CR-PCAT questionnaire took about 5 to 7 minutes to administer. The tool greatly reduces the burden of patients and interviewers while insuring the key information was captured. CR-PCAT can be applied in periodic evaluation activities to assess the latest performance of primary care provided by CHCs. Also, since the tool is readily linked to specific implementations of primary care, it can be used to assess the effectiveness of policy changes on the delivery of care from both CHCs and district levels. On condition that primary care is provided by GPs in China, CR-PCAT can also serve as a part of the performance evaluation tools of GPs to provide evidence for further improvement.

In addition, from the primary care achievements of three cities, low scores of the overall primary care quality and each dimension suggest that primary care in China is far from perfect. The low scores of each dimension were consistent with our previous studies, which focused specifically on continuity or coordination of care [47–49]. Besides, significant differences of scale scores between three cities were observed, implying that there might be system or organizational level factors that cause the differences [50]. For example, the score of first contact utilization in Dongguan was the highest among the three cities, which might imply the effects of gatekeeping system; and Shenzhen achieved the highest scores on the scale of coordination, indicating that patients experience better referral services when specialist care is needed, which may be due to the reason that CHCs in Shenzhen were affiliated to hospitals while the CHCs in the other two cities were independent of hospitals.

### 4.2. Limitations

Potential limitations of the study are discussed as follows. First, the CR-PCAT is a very short version of the original measure tool and the development and adjustment of the tool were straightforward. Only quantitative analyses were conducted in the study, which might imply that qualitative methods such as focus groups or in-depth interviews with patients might be necessary to fully explore their perception of primary care. Also, CR-PCAT does not measure disease specific quality of care, but rather general experience of primary care. Further research could combine CR-PCAT with disease specific quality indicators to have more extensive representation.

Secondly, the CR-PCAT was developed based on CHCs samples obtained in Guangdong province, where local people mainly speak Cantonese, suggesting that the linguistic habits were unique. If the tool were to be used in other provinces in China where there were distinct differences between the dialects, slight revisions of the wording were deemed necessary.

Thirdly, the measurement of primary care achievement was based on patients' self-report. While this may be the best way to ascertain individual experience, it is subject to recall and response bias. Nevertheless, one-to-one interviews were undertaken in this study in an attempt to minimize the bias. In addition, criterion-related validity (or more specifically concurrent validity) and stability were not tested due to time and economic reasons; however, the tool will be applied in more regions and populations in the future, the results of which could add to the validation evidence of CR-PCAT.

Finally, as the CR-PCAT only measured adult patient's perception of primary care, which was adapted from one part of the original methodology, our future research would center on developing rapid assessment tools for providers and children.

## 5. Conclusion

CR-PCAT appears to be a valid and effective tool to capture reliable performance of four core dimensions of primary care in China. Because of its simplicity and easy administration, it is a feasible and practical tool that can be used in CHCs' daily administration, performance evaluation and monitoring, and policy assessments. The poor performance of primary care highlights the urgency of recognition and understanding of its core dimensions and the development of corresponding policies in the future to strengthen them in China. The next phase of the study will focus on identifying characteristics at the organization and health care system levels that account for the observed differences in primary care performance.

## Figures and Tables

**Table 1:** Sociodemographic characteristic of the study subjects.

	Total (%)
	1465

Gender	
Male	574 (39.2)
Female	889 (60.7)

Age (years)	
<40	616 (42.0)
40–65	600 (41.0)
>65	249 (17.0)

Household registration	
Resident population	715 (48.8)
Temporary resident population	729 (49.8)
Floating population	17 (1.2)

Household income (yuan/month)	
<5000	459 (31.4)
5000–10000	496 (33.9)
>10000	291 (19.9)

Education	
High school or lower	1006 (69.1)
Vocational/associate	291 (20.0)
College or higher	159 (10.9)

Employment	
Employed (including part-time)	798 (54.6)
Unemployed	224 (15.3)
Retired	423 (29.0)
In school	16 (1.1)

Medical insurance	
Urban-employees	629 (42.9)
Urban-citizens	524 (35.8)
Out-of-pocket expenditure	312 (21.3)

Period of time since the first visit	
Less than 1 year	254 (17.3)
1-2 years	229 (15.6)
3–5 years	305 (20.8)
More than 5 years	673 (45.9)

Number of PCP visits in the past year	
<3	391 (26.7)
3–5	403 (27.5)
6–15	440 (30.1)
>15	229 (15.7)

Specialist visits in the past year	
No	683 (46.6)
Yes	782 (53.4)

Contracted PCP	
No	1334 (91.1)
Yes	131 (8.9)

Chronic disease	
No	853 (58.2)
Yes	612 (41.8)

Self-perceived health status	
Excellent	101 (6.9)
Very good	568 (38.8)
Good	455 (31.1)
Fair	304 (20.8)
Poor	37 (2.5)

**Table 2:** The characteristics of each city and CHC.

Cities	CHCs	Owned status	Gatekeeping	Operational mode	Sample size
Guangzhou	Hong shan	Private-owned	No	Independent	292
	Sha yuan	Government-owned	No	Independent	208
	Huang hua gang	Government-owned	No	Independent	198

Shenzhen	Niu hu	Government-owned	Yes	Affiliated to public hospital	183
	Yi kang yuan	Government-owned	Yes	Affiliated to public hospital	95
	Liu tang	Government-owned	Yes	Affiliated to public hospital	90

Dongguan	Liao bu	Government-owned	Yes	Independent	199
	Da lang	Government-owned	Yes	Independent	200

**Table 3:** Factor analysis result with five factors retained.

Final rotated factor loadings					
Items	Factor 1	Factor 2	Factor 3	Factor 4	Factor 5
(Q9) When you have a new health problem, do you go to your PCP before going somewhere else?					0.7166
(Q10) When you need a regular general checkup, do you go to your PCP before going somewhere else?					0.7052
(Q11) When you have to see a specialist, does your PCP have to approve or give you a referral?					0.4621

(Q13) When your PCP is *open*, can you get advice quickly over the phone if you need it?				0.8661	
(Q14) When your PCP is *closed*, can you get advice quickly over the phone if you need it?				0.8801	

(Q15) When you go to your PCP's, are you taken care of by the *same* doctor or nurse each time?	0.8111				
(Q16) If you have a question, can you call and talk to *the doctor or nurse who knows you best*?	0.7755				
(Q17) Does your PCP know what problems are most important to you?	0.7318				

(Q18) When you have a mental or emotional problems, do you talk to your PCP?			0.5721		
(Q19) Does your PCP talk about healthy diet, adequate sleep, appropriate exercise and so forth with you?			0.6509		
(Q20) Does your PCP recommend you to have a regular checkup?			0.6917		
(Q21) Generally, how much time will your PCP spend talking to you?			0.5588		

(Q24) Did your PCP discuss with you different places you could have gone to get help with that problem?		0.7305			
(Q26) Did your PCP or someone working with your PCP help you make the appointment for that visit?		0.7278			
(Q27) After you went to the specialist or special service, did your PCP talk with you about what happened at the visit?		0.5421			

Eigenvalue	2.0856	1.8367	1.8277	1.6934	1.3925
Variance (%)	13.90	12.24	12.18	11.29	9.28
Accumulative variance (%)	13.90	26.15	38.33	49.62	58.91

**Table 4:** Item statistics and internal consistency reliability of original primary care scales and revised scales.

Primary care scale items	Reliability-alpha Sample size for revised scale	Item mean	Item SD	Orig item- total corr	Rev item- total corr
First contact utilization	Orig 3-item *a* = 0.38 Rev 3-item *a* = 0.38; *n* = 1465				

(Q9) When you have a new health problem, do you go to your PCP before going somewhere else?		3.63	0.66	0.56	0.56
(Q10) When you need a regular general checkup, do you go to your PCP before going somewhere else?		2.84	1.24	0.75	0.75
(Q11) When you have to see a specialist, does your PCP have to approve or give you a referral?		2.02	1.23	0.70	0.70

First contact accessibility	Orig 3-item *a* = 0.24 Rev 2-item *a* = 0.74; *n* = 1465				

(Q12) Once you get there, do you have to wait more than 15 minutes before you are checked by the doctor or nurse?		2.74	0.88	0.77	Deleted
(Q13) When your PCP is *open*, can you get advice quickly over the phone if you need it?		1.14	0.42	0.57	0.91
(Q14) When your PCP is *close*, can you get advice quickly over the phone if you need it?		1.09	0.36	0.56	0.88

Ongoing care	Orig 3-item *a* = 0.72 Rev 3-item *a* = 0.72; *n* = 1465				

(Q15) When you go to your PCP's, are you taken care of by the *same* doctor or nurse each time?		2.19	1.13	0.83	0.83
(Q16) If you have a question, can you call and talk to *the doctor or nurse who knows you best*?		3.00	1.10	0.80	0.80
(Q17) Does your PCP know what problems are most important to you?		2.40	0.93	0.78	0.78

Comprehensiveness	Orig 4-item *a* = 0.58 Rev 4-item *a* = 0.58; *n* = 1465				

(Q18) When you have a mental or emotional problems, do you talk to your PCP?		1.31	0.67	0.63	0.63
(Q19) Does your PCP talk about healthy diet, adequate sleep, appropriate exercise and so forth with you?		2.06	0.83	0.72	0.72
(Q20) Does your PCP recommend you to have a regular checkup?		1.78	0.89	0.72	0.72
(Q21) Generally, how much time will your PCP spend talking to you?		2.00	0.72	0.59	0.59

Coordination	Orig 4-item *a* = 0.54 Rev 3-item *a* = 0.57; *n* = 783				

(Q24) Did your PCP discuss with you different places you could have gone to get help with that problem?		1.61	0.90	0.68	0.74
(Q25) Did your doctor write down any information for the specialist about the reason for the visit?		2.76	1.27	0.70	Deleted
(Q26) Did your PCP or someone working with your PCP help you make the appointment for that visit?		1.37	0.76	0.65	0.77
(Q27) After you went to the specialist or special service, did your PCP talk with you about what happened at the visit?		1.76	0.89	0.60	0.70

Derivative dimensions	Orig 6-item *a* = 0.68 All rev items were deleted				

(Q28) Does your doctor ask your ideas and opinions when they are planning treatment/care for you or a family member?		1.93	0.98	0.37	Deleted
(Q29) Has your doctor asked about illness or problems that might run in your family?		1.54	0.77	0.46	Deleted
(Q30) Would anyone at doctor s office ever make home visits?		1.11	0.38	0.40	Deleted
(Q31) Does your doctor know about health problems of your neighborhood?		1.37	0.63	0.49	Deleted
(Q32) How does (Doctor/Place P) get opinions/ideas from people that will help them provide better health care?		1.26	0.58	0.37	Deleted
(Q33) Would you recommend your doctor to a friend or relative?		1.97	0.99	0.31	Deleted

**Table 5:** Likert scaling assumptions using revised items.

Scale	Range of item-scale correlations (assumptions 1, 4)	Item scaling tests (assumption 2) Success/total scaling success rate	Measures of equal item variance (assumption 3)		Cronbach's alpha (assumption 5)
			Scott's homogeneity	Intraclass correlation	
First contact utilization	0.56–0.75	15/15	0.20	0.17	0.38
First contact accessibility	0.87–0.91	10/10	0.60	0.58	0.74
Ongoing care	0.78–0.83	15/15	0.47	0.47	0.72
Comprehensiveness	0.67–0.77	20/20	0.26	0.30	0.58
Coordination of services	0.70–0.77	15/15	0.32	0.26	0.57

**Table 6:** Estimates of central tendency and dispersion of PCAT scales.

	First contact utilization	First contact accessibility	Ongoing care	Comprehensiveness	Coordination
Number of items	3	2	3	4	3
Mean	8.49	2.23	7.59	7.15	4.73
25th percentile	7.00	2.00	6.00	6.00	3.00
50th percentile	9.00	2.00	7.50	7.00	4.00
75th percentile	10.00	2.00	10.00	8.00	6.00
Observed range	3.0–12.0	2.0–8.0	3.0–12.0	4.0–16.0	3.0–12.0
SD	2.16	0.70	2.54	2.09	1.87
Skewness	−0.03	3.86	−0.15	0.88	1.17
Kurtosis	2.31	20.79	2.17	3.95	4.17

**Table 7:** Comparison of internal consistency reliability and interfactor correlation.

Factor	Cronbach's alpha	Interfactor correlations				
		First contact utilization	First contact accessibility	Ongoing care	Comprehensiveness	Coordination
Without coordination items (*n* = 1465)						
First contact utilization	0.38	1.00				
First contact accessibility	0.74	0.00	1.00			
Ongoing care	0.72	0.03	0.13	1.00		
Comprehensiveness	0.58	0.01	0.27	0.32	1.00	
With coordination items (*n* = 783)						
First contact utilization	0.38	1.00				
First contact accessibility	0.74	0.03	1.00			
Ongoing care	0.72	0.07	0.11	1.00		
Comprehensiveness	0.58	0.12	0.21	0.29	1.00	
Coordination	0.57	0.23	0.32	0.11	0.27	1.00

**Table 8:** Primary care quality scores in three cities.

Scale	Guangzhou (SE)	Dongguan (SE)	Shenzhen (SE)
First contact utilization	2.63 (0.02)^*∗*#^	3.10 (0.04)^&^	2.92 (0.04)
First contact accessibility	1.67 (0.01)^*∗*#^	1.54 (0.02)^&^	1.76 (0.02)
Ongoing care	2.70 (0.03)^*∗*#^	2.45 (0.05)	2.29 (0.04)
Comprehensiveness	1.87 (0.02)^*∗*^	1.60 (0.02)^&^	1.83 (0.03)
Coordination of services	1.57 (0.02)^*∗*#^	1.80 (0.02)^&^	2.17 (0.03)
Total	2.09 (0.01)^#^	2.10 (0.02)	2.19 (0.02)

(a) Significance is indicated at *P* < 0.05, based on one-way ANOVA (Bonferroni).

(b) ^*∗*^Indicating significant differences between Guangzhou and Dongguan.

(c) ^#^Indicating significant differences between Guangzhou and Shenzhen.

(d) ^&^Indicating significant differences between Dongguan and Shenzhen.

**Table 9:** Specific item changes compared to the original tool.

Scales	Items contained in original tool	Items retained in preliminary tool	Comments
First contact utilization	(B1) When you need a regular general checkup, do you go to your PCP before going somewhere else?	(Q9) When you have a new health problem, do you go to your PCP before going somewhere else?	All original items in “first contact utilization” scale were retained; slight change was made on item order
	(B2) When you have a new health problem, do you go to your PCP before going somewhere else?	(Q10) When you need a regular general checkup, do you go to your PCP before going somewhere else?	
	(B3) When you have to see a specialist, does your PCP have to approve or give you a referral?	(Q11) When you have to see a specialist, does your PCP have to approve or give you a referral?	

First contact accessibility	(C3) When your PCP is open and you get sick, would someone from there see you the same day?	(Q12) Once you get there, do you have to wait more than 15 minutes before you are checked by the doctor or nurse?	(C3) was replaced by (Q12) because almost all of the patients can be seen the same day when they turn to a CHC in China; (C7) was deleted because most of the CHCs in China do not offer medical services at night
	(C4) When your PCP is open, can you get advice quickly over the phone if you need it?	(Q13) When your PCP is open, can you get advice quickly over the phone if you need it?	
	(C5) When your PCP is closed, is there a phone number you can call when you get sick?	(Q14) When your PCP is closed, can you get advice quickly over the phone if you need it?	
	(C7) When your PCP is closed and you get sick during the night, would someone from there see you that night?		

Ongoing care	(D1) When you go to your PCP's, are you taken care of by the same doctor or nurse each time?	(Q15) When you go to your PCP's, are you taken care of by the same doctor or nurse each time?	(D7) was deleted due to its unfitness for the local context
	(D4) If you have a question, can you call and talk to the doctor or nurse who knows you best?	(Q16) If you have a question, can you call and talk to the doctor or nurse who knows you best?	
	(D7) Does your PCP know you very well as a person, rather than as someone with a medical problem?	(Q17) Does your PCP know what problems are most important to you?	
	(D8) Does your PCP know what problems are most important to you?		

Comprehensiveness (service available)	(G2) Immunizations (shots)	(Q18) When you have a mental or emotional problems, do you talk to your PCP?	(G2), (G6), and (G10) were deleted because they are the regular healthcare services provided by CHCs
	(G6) Family planning or birth control methods		
	(G8) Counseling for mental health problems		
	(G10) Sewing up a cut that needs stitches		

Comprehensiveness (service provided)	(H1) Advice about healthy foods and unhealthy foods or getting enough sleep	(Q19) Does your PCP talk about healthy diet, adequate sleep, appropriate exercise and so forth with you?	(H1), (H5) were combined; (H2), (H4), and (H7) were deleted due to their unfitness for the local context
	(H2) Home safety, like getting and checking smoke detectors and storing medicines safely	(Q20) Does your PCP recommend you to have a regular checkup?	
	(H4) Ways to handle family conflicts that may arise from time to time	(Q21) Generally, how much time will your PCP spend talking to you?	
	(H5) Advice about appropriate exercise for you		
	(H7) Checking on and discussing the medications you are taking		

Coordination	(E8) Did your PCP discuss with you different places you could have gone to get help with that problem?	(Q24) Did your PCP discuss with you different places you could have gone to get help with that problem?	All original items in “coordination” scale were retained; slight change was made on item order
	(E9) Did your PCP or someone working with your PCP help you make the appointment for that visit?	(Q25) Did your doctor write down any information for the specialist about the reason for the visit?	
	(E10) Did your PCP write down any information for the specialist about the reason for the visit?	(Q26) Did your PCP or someone working with your PCP help you make the appointment for that visit?	
	(E12) After you went to the specialist or special service, did your PCP talk with you about what happened at the visit?	(Q27) After you went to the specialist or special service, did your PCP talk with you about what happened at the visit?	

Coordination (information systems)	(F1) When you go to your PCP, do you bring any of your own medical records, such as shot records or reports of medical care you had in the past?		(F1), (F2), and (F3) were all deleted due to their unfitness for the local context
	(F2) Could you look at your medical record if you wanted to?		
	(F3) When you go to your PCP, is your medical record always available?		

Family centredness	(I1) Does your PCP ask you about your ideas and opinions when planning treatment and care for you or a family member?	(Q28) Does your doctor ask your ideas and opinions when they are planning treatment/care for you or a family member?	(I3) was deleted due to its unfitness for the local context
	(I2) Has your PCP asked about illnesses or problems that might run in your family?	(Q29) Has your doctor asked about illness or problems that might run in your family?	
	(I3) Would your PCP meet with members of your family if you thought it would be helpful?		

Community orientation	(J1) Does anyone at your PCP's office ever make home visits?	(Q30) Would anyone at doctor s office ever make home visits?	All original items in “community orientation” scale were retained
	(J2) Does your PCP know about the important health problems of your neighborhood?	(Q31) Does your doctor know about health problems of your neighborhood?	
	(J3) Does your PCP get opinions and ideas from people that will help to provide better health care?	(Q32) Does your PCP get opinions/ideas from people that will help to provide better health care?	

Cultural competence	(K1) Would you recommend your PCP to a friend or relative?	(Q33) Would you recommend your doctor to a friend or relative?	(K2) and (K3) were deleted due to their unfitness for the local context
	(K2) Would you recommend your PCP to someone who does not speak English well?		
	(K3) Would you recommend your PCP to someone who uses folk medicine, such as herbs or homemade medicines, or has special beliefs about health care?		

## Abbreviations

PCAT: Primary Care Assessment Tool
PCAT-AE: Primary Care Assessment Tool-Adult Edition
CR-PCAT: Chinese Rapid Primary Care Assessment tool
IOM: Institute of Medicine
GP: General practitioner
CHC: Community health center.
